# The urgency of Marburg therapeutics: preventing local outbreaks from the potential global spread

**DOI:** 10.3389/fmicb.2024.1378314

**Published:** 2024-07-01

**Authors:** Tarun Kumar Suvvari, Ahmed Mahal, Venkataramana Kandi, Hadil Faris Alotaibi, Snehasish Mishra, Ranjan K. Mohapatra

**Affiliations:** ^1^Department of Medicine, Rangaraya Medical College, Kakinada, India; ^2^Department of Medicine, Dr. YSR University of Health Sciences, Vijayawada, India; ^3^Department of Medicine, Squad Medicine and Research (SMR), Vizag, India; ^4^Department of Medical Biochemical Analysis, College of Health Technology, Cihan University-Erbil, Erbil, Kurdistan Region, Iraq; ^5^Department of Microbiology, Prathima Institute of Medical Sciences, Karimnagar, Telangana, India; ^6^College of Pharmacy, Princess Nourah Bint Abdulrahman University, Riyadh, Saudi Arabia; ^7^School of Biotechnology, Campus-11, KIIT Deemed-to-be-University, Bhubaneswar, Odisha, India; ^8^Department of Chemistry, Government College of Engineering, Keonjhar, Odisha, India

**Keywords:** Marburg (MARV), Marburg research gap, vaccines and antiviral, transmission, prevention

## Introduction

Emerging infectious diseases like the COVID-19 swiftly spread to ultimately become the largest reported pandemic ever from a mere local zoonotic event. Thus, the need to invest more in research and innovation in therapeutics and preventives for the numerous rare, emerging and reemerging diseases including the Marburg virus disease (MVD) was felt (Srivastava S. et al., 2023). The highly pathogenic Marburg virus (MARV), a member of the *Filoviridae* family, causes severe haemorrhagic fever in humans and nonhuman primates, with appreciably high mortality rate (Mohapatra et al., 2022; Srivastava S. et al., 2023). The MVD mortality was between 23 and 90% with an average 50% fatality rate, as per the WHO (https://www.who.int/news-room/fact-sheets/detail/marburg-virus-disease). Emerging infectious diseases like COVID-19 even in the face of extending various tightened and heightened public health measures and the geographical limitations, the potential for unexpected spillover events is not ruled out (Islam, 2023; Islam et al., 2023a). Coupled with the lack of effective therapeutics for the highly fatal Filovirus with high mortality rate of Marburg virus (MARV), the global focus on developing therapeutics and preventives against such emerging pathogens has heightened.

MVD outbreak is devastating round year mainly in the African continent, with high CFR. Conducting surveys and research studies on the earlier outbreaks and the available data regarding MVD outbreaks is therefore important. The discovery of the prophylactics and therapeutics (vaccines and antiviral drugs) to control the emergence of MVD need to be prioritized due to the dearth of specific treatment regime (Mohapatra et al., 2023). Earlier studies opined that clinical investigations of MVD alone are not enough. Existing knowledge about host-virus interactions that could facilitate designing and developing vaccines or therapeutics to control MVD seems to be shallow (Islam et al., 2023b). An earlier study has identified GP and VP40 matrix proteins as the most potent antigenic viral protein candidates to develop chimeric subunit vaccines (Hasan et al., 2019). Considering the need to bridge the knowledge gap about its pathogenicity, its potential aerosolised transmission, and the lack of immunological and pharmacological therapeutic measures, extensive research on MVD therapeutics is urgently required (Mohapatra et al., 2022). The role of bats as natural reservoirs for an array of Filoviruses including the MARV emphasizes the need to understand spillover dynamics and the zoonotic transmission route (Sah et al., 2022). The threats of transmission of Filoviruses through aerosols further accentuate the urgency of an effective therapeutic intervention to mitigate the risk (Mekibib and Ariën, 2016).

## Discussion

The global urgency for Marburg therapeutics is evident in the context of the emerging infectious diseases (Cross et al., 2022). A bibliometric study on global research trends of the emerging pathogens including Marburg virus highlights the need for a heightened awareness and preparedness to address the challenges that is imminent (Sweileh, 2017). While it is acknowledged that Filoviruses (including Ebola and Marburg viruses) are involved in relatively few outbreaks as compared to other such infectious diseases prevalent especially in Africa, devastating impact of the outbreaks on human health necessitates the need for prophylactic and therapeutic measures in place (Sweileh, 2017; Cross et al., 2022).

Developing antiviral drugs is crucial to tackle pathogenic viruses. Currently, there is no approved therapeutics against MVD, highlighting the need for extensive research. Nonclinical studies have shown promising efficacy of BCX4430 against Marburg, indicating its potential repositioning as a broad-spectrum antiviral (Taylor et al., 2016). The efficacy of another antiviral drug Galidesivir was tried on MARV-challenged cynomolgus macaques, which showed decreased viraemia levels and increased survival rates (Kortepeter et al., 2020). Monoclonal antibodies (mAbs) were effective against non-human primates (Mehedi et al., 2011). A mAb MR 191-N was also tried in the lab, and results are yet to be disclosed (Albakri et al., 2023). REGNEB3, ZMapp and mAb114 that successfully treated Ebola may be considered as a good option against MVD too (Kortepeter et al., 2020). A mAb and remdesivir combined therapy is also be a promising therapeutic option against Filovirus diseases like MVD (Cross et al., 2021). Visual imaging of molecular structures of MARV proteins is essential to develop effective antiviral drugs, which emphasizes the significance of carrying out such research. As an innovative strategy in antiviral drug discovery, *in silico* studies and molecular docking approaches were employed to identify potential inhibitors and therapeutic targets against MARV (Akash et al., 2023). Pyronaridine, tilorone and quinacrine were identified as the Ebola and Marburg virus inhibitors. Developing lipid-encapsulated siRNA as MARV therapeutant demonstrates the diverse approaches in exploring effective therapeutics (Ursic-Bedoya et al., 2014). NP718m, a siRNAs, was tested on MARV-infected guinea pig (Kortepeter et al., 2020). The validated experimental results of trials on the non-human primate models have not been tried in humans, neither the validated data is published and made public.

The need for extensive research to develop effective countermeasures against Marburg disease is urgently felt in the absence of clinically approved vaccines or therapeutic agents. A recent study assessed controlling the MARV outbreak using mathematical models, and the results showed that vaccination could be the best strategy (Qian et al., 2023). Innovative research efforts need to focus on developing vaccines and antiviral drugs as effective interventions. Currently, Favipiravir (T-705) is used to treat influenza and also in phase II clinical trials for to treat Ebola (Albakri et al., 2023). The potential of broad-spectrum antiviral agents like T-705 against Filoviruses that include Marburg virus highlights the importance of exploring diverse therapeutic strategies (Zhu et al., 2018; Srivastava S. et al., 2023). The discovery and development of inhibitors with broad-spectrum antiviral properties against haemorrhagic fever like that by Ebola and MARV is yet another innovative approach (Mohr et al., 2015).

Given the highly pathogenic nature of MARV infection and its reemergence often spreading to non-endemic regions with high (>80%) human mortality, a vaccine against it is an urgent need. As a maiden attempt, clinical trials of a modified chimpanzee Adenovirus 3 (cAd3) coded with MARV Angola glycoprotein as a vaccine was tried on humans (Hamer et al., 2023). It was developed by the national institute of allergy and infectious diseases (NIAID) researchers of the national institutes of health (NIH), USA. The Phase-1 vaccine trial was conducted on 40 healthy adults who were given a single intramuscular shot of the vaccine with 1X10^11^ protein particle units (pu). Although no serious vaccine side-effects were evident, vaccinated individuals showed mild effects like pain and tenderness at the injection site (68%), headache (43%), malaise (45%) and myalgia (35%). It was found to be sufficiently (95%) immunogenic with neutralizing antibody being observed 4 weeks after immunization that remained detectable for up to 48 weeks among 70% of those vaccinated.

Another MARV vaccine candidate that used MVA-BN-Filo (containing both MARV and Ebola virus antigens) with MARV-DNA as the vector is currently undergoing Phase-1 trial (Finch et al., 2022). Although MVA-BN-Filo/Mvabea vaccine was granted approval by the European Medical Agency (EMA) against Ebola virus infection (https://www.who.int/news-room/fact-sheets/detail/marburg-virus-disease), none of the vaccine candidate has entered advance clinical trial stages as yet. Another MARV vaccine trial was co-initiated by Makerere University Walter Reed Project (MUWRP) and Sabin Vaccine Institute, supported by the Biomedical Advanced Research and Development Authority (BARDA), USA. Its Phase-2 trial was being carried out in Uganda and Kenya among 125 healthy volunteers of 18–50 years and 51–70 years age groups. Vaccine candidates for MARV have been constructed using platforms like DNA plasmids, vector virus carriers like recombinant vesicular stomatitis virus (VSV), adenovirus and virus-like particles (VLP) among others. Among these candidates, the MARV DNA vaccines are noted to have low immunogenicity that last for a short period when human tested (Dulin et al., 2021). However, trials conducted on non-human primates (NHP) showed promising results with a majority of the vaccine candidates.

Evolving biodefense field emphasizes on the need to develop diagnostics and therapeutics to combat deadly the emerging and reemerging infectious diseases, highlighting the need for a concerted global effort to address the public health threat posed by Marburg virus (Janik et al., 2020). Global urgency to counter MARV necessitates collaborative research efforts and standardizing the developmental approaches. Standardized assays and preclinical trials could accelerate scoping therapeutic interventions and streamlining product development initiatives. As MARV is a risk group-4 (RK-4) pathogen, a major drawback of MARV disease diagnosis is the need for biosafety level-4 (BSL-4) containment for specimen handling and to carryout laboratory tests. A RK-4 pathogen is highly pathogenic without a vaccine, specific treatment modality, and managing and preventing the infection. Thus, most low- and middle-income African nations where MARV is endemic could not afford it as establishing its research, development and diagnosis need huge finances and logistics (Miraglia, 2019; ECDC, 2023).

Musoke, Angola, Ci67, Ozolin, Popp, Ratayczak and Voege are various MARV variants. These have a majority of common proteins with minor genomic differences. MARV has many proteins that are immunogenic and elicit neutralizing antibody responses (Gordon et al., 2019). These viral protein antigens include RNA-associated nucleoprotein (NP), viral protein30 (VP30), VP35, nucleocapsid protein [L-polymerase (L)], matrix protein (VP40, VP24) and surface glycoprotein (GP) (Islam et al., 2023b). Among all such viral proteins, glycoproteins seem variant-specific.

Egyptian fruit-bat (*Rousettus aegyptiacus*) is the natural hosts and primary reservoirs of MARV, which infects both animals and humans. After its first reports from Germany and Serbia in 1967, many outbreaks were recorded majorly in Africa (Srivastava D. et al., 2023). As the mechanism of bat-bat transmission of MARV as also to other animals is yet unclear, a prudent approach to avoid bat-human contracting of MVD is by reduce anthropogenic activities on the mining areas or caves that are the primary habitat of fruit-bat (Mohapatra et al., 2022). Tourists and researchers visiting fruit-bat infested mines must follow appropriate safety measures. Pig acts as a significant intermediate/amplification host in transmitting many wildlife viruses (like Nipah virus, vesicular stomatitis virus, Eastern equine encephalitis virus, Japanese encephalitis virus, Reston Ebola virus, etc.) in humans (Glud et al., 2021). Pigs as hosts for novel coronaviruses is also established (McLean and Graham, 2022). Pig-human transmission of zoonotic viruses are usually linked to occupational exposure. One Ebola virus member was not only capable of infecting pigs but also being transmitted between pigs and co-housed non-human primates in indirect contact (Lewis et al., 2024), suggesting the potential role as amplifying host in MVD outbreaks (WHO, 2022; Zhao et al., 2022). Pig farm owners in Africa must take precautions to prevent pigs from contracting the disease. Pigs should be routinely vaccinated to safeguard human health (Dhama et al., 2022). Many other domesticated animals reportedly do not have any link in Filovirus outbreaks.

**Figure 1:**
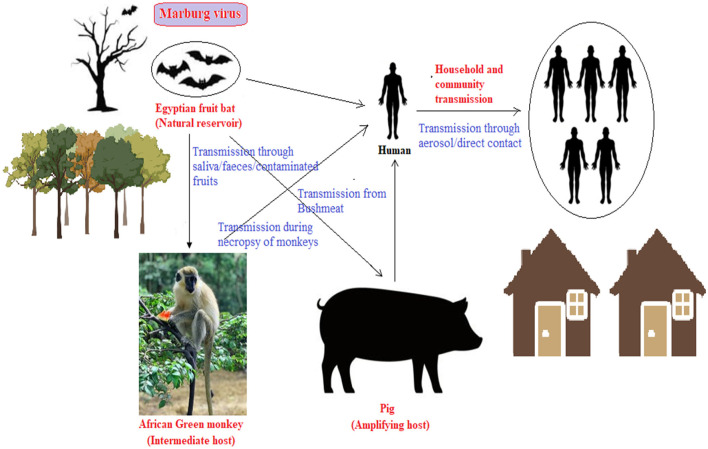
Human-animal zoonosis interface for human spillover of MARV (illustrates the presence of the virus in the environment and its role in potential animal-human MARV transmission routes, highlighting the need of the One-Health approach. Monkey and pig may be intermediate and amplifying hosts, while fruit bat is natural reservoir. Humans could contract the virus through direct or indirect contact with the infected animals, their fluids or through close contact with other infected individuals).

MVD is classified as a neglected tropical disease by the WHO primarily due to lack of attention, lack of funds, and fringe research as compared to many other infectious diseases. Being reported as small outbreaks, the available data is inadequate. MARV is noted to have pandemic potential mostly attributed to the fact that it has spread from its endemic African region to newer non-endemic geographical areas, like Europe. The transition of MARV from local outbreaks to the global pandemic potential underscores a need for extensive vaccines and therapeutics research and development. Collaborative efforts to decipher and bridge the gaps following the “One Health” model are urgent. Focusing on the human-animal interface (natural reservoir, intermediate hosts, possible amplifier hosts, etc.) in the endemic regions is suggested (Figure 1). Challenges posed by the lack of knowledge on the pathogenicity and transmission dynamics of MARV necessitate global collaborative research and innovative therapeutic interventions especially in the face of dearth of approved therapeutics (Srivastava D. et al., 2023). Collective research efforts from diverse expertise to develop broad-spectrum vaccines and antiviral agents could contribute to effectively address the MARV public health threat. Further, lethal viruses like the MARV can be handled only in a Biosafety Level 4 (BSL-4) complied laboratory which are far and few. Hence, laboratories with basic and applied research capabilities are incapable of handling and developing prophylactics and therapeutics against this lethal MARV.

A multipronged approach to tackle a possible global MVD outbreak in future needs to be initiated with comprehensive and coordinated implementation of the same. Global public health infrastructure for early detection, rapid response and effective control measures implementation need to be strengthened, particularly in resource-limited regions. It includes establishing a robust surveillance networks, supporting the local healthcare system and personnel training. Research and innovation to create and stock effective diagnostics, therapeutics and prophylactics could be accelerated, including mass vaccination or ring vaccination programmes for the target groups. Public and also the healthcare workers' awareness and education campaign about MVD particularly in regions that are at greater risk, measures to practice hygiene and sanitation and seeking healthcare promptly with the initial symptoms are equally crucial. Proactive, robust and effective planning collaboratively by the researchers, public health experts, policymakers and biologists are necessary to design suitable strategies for the preparedness to counter epidemics in future such as that of MVD (Islam et al., 2023b). Literature suggests that circumspection, wastewater monitoring and prognosis of an outbreak are crucial in the process. Thus, employing wastewater-based surveillance with serological and molecular epidemiological investigations especially in the suspected regions would help. International networking to foster information sharing, mobilize the resources and coordinate response efforts beyond the international borders is vital.

## Author contributions

TS: Conceptualization, Data curation, Writing—original draft. AM: Data curation, Writing—original draft. VK: Data curation, Writing—original draft. HA: Writing—original draft. SM: Writing—review & editing. RM: Supervision, Methodology, Writing—review & editing.
